# Oligomerization of *Drosophila* Nucleoplasmin-Like Protein is required for its centromere localization

**DOI:** 10.1093/nar/gky988

**Published:** 2018-10-24

**Authors:** Eduard Anselm, Andreas W Thomae, A Arockia Jeyaprakash, Patrick Heun

**Affiliations:** 1 Max Planck Institute of Immunobiology and Epigenetics, Freiburg, Germany; 2 Faculty of Biology, Albert Ludwigs Universität Freiburg, Freiburg, Germany; 3 Wellcome Trust Centre for Cell Biology, Edinburgh, UK; 4 Biomedical Center, Core Facility Bioimaging, Ludwig-Maximilians-Universität München, Planegg-Martinsried, Germany

## Abstract

The evolutionarily conserved nucleoplasmin family of histone chaperones has two paralogues in *Drosophila*, named Nucleoplasmin-Like Protein (NLP) and Nucleophosmin (NPH). NLP localizes to the centromere, yet molecular underpinnings of this localization are unknown. Moreover, similar to homologues in other organisms, NLP forms a pentamer *in vitro*, but the biological significance of its oligomerization has not been explored. Here, we characterize the oligomers formed by NLP and NPH *in vivo* and find that oligomerization of NLP is required for its localization at the centromere. We can further show that oligomerization-deficient NLP is unable to bind the centromeric protein Hybrid Male Rescue (HMR), which in turn is required for targeting the NLP oligomer to the centromere. Finally, using super-resolution microscopy we find that NLP and HMR largely co-localize in domains that are immediately adjacent to, yet distinct from centromere domains defined by the centromeric histone dCENP-A.

## INTRODUCTION

Histone chaperones perform crucial functions in chromatin biology. The first histone chaperone has been isolated from *Xenopus* egg extracts and due to its high abundance in the egg nucleoplasm was termed ‘nucleoplasmin’ (1–3). Nucleoplasmin is an acidic protein that is pentameric in solution and, as a histone chaperone, can directly bind to histones and assemble nucleosomes in the presence of DNA *in vitro* (1,2). *In vivo*, nucleoplasmin is required for sperm chromatin remodelling after fertilization of *Xenopus* eggs (4,5). Homologues of nucleoplasmin have been found in other vertebrates and in invertebrates (6). Considerable attention has been devoted towards understanding the human homologue Nucleophosmin 1 (NPM1). NPM1 localizes predominantly to the nucleolus and functions in a multitude of cellular processes, including ribosome biogenesis, DNA repair, transcription and centrosome duplication (7,8). Some of the interest in NPM1 stems from the fact that genetic alterations of the NPM1 gene are associated with haematological cancer, while overexpression of NPM1 has been found in a variety of other cancers (9). Therefore, NPM1 might represent a potential target for cancer therapy (10).

Common to members of the nucleoplasmin protein family is a structured N-terminal ‘core’ domain and a flexible C-terminal ‘tail’ domain (11). Crystal structures of the core domains of several nucleoplasmin homologues have been characterized and revealed that each monomer consists of an eight-stranded β-barrel and five monomers associate to form a cyclic pentamer (12–16). In some instances, this pentamer has been found to dimerize to form a decamer (12,14,16). Oligomerization of human NPM1 has been found to be important for different aspects of its functions, including nucleolar localization and nucleosome assembly (17–20). Thus, insights into the formation of oligomers by nucleoplasmin homologues in other organisms is important for a thorough understanding of their function.

In *Drosophila*, two nucleoplasmin homologues are present, termed Nucleoplasmin-Like Protein (NLP, formerly also called p22 (21) or CRP1 (22)) and Nucleophosmin (NPH) (6,23). Similar to other nucleoplasmin homologues, the crystal structure of NLP has revealed a pentamer with five monomers arranged in a cyclic manner (13). Oligomeric forms of NLP have been detected in embryonic extracts after chemical cross-linking (22), but the functional significance of NLP oligomers *in vivo* remains unknown.

Similar to *Xenopus* nucleoplasmin, NLP and NPH are both implicated in sperm chromatin remodelling upon fertilization of the oocyte (23). In addition, NLP contributes to pairing of homologous chromosomes (24) and is required for the clustering of centromeres around the nucleolus during interphase (25). NLP localizes to the nucleoplasm, is excluded from the nucleolus and concomitant with its proposed centromeric function, distinctively at the centromere throughout interphase in somatic cells (21,25,26).

The centromere is an essential chromosomal domain that is located at the primary constriction site of chromosomes and required for the attachment of the microtubules for chromosome segregation (27). Similar to most eukaryotes, the centromere in *Drosophila* is defined by the presence of a specific histone H3 variant, termed centromere protein A (CENP-A; dCENP-A in *Drosophila*) (28). Proteins that have been found to localize to the centromeric region in *Drosophila* include Hybrid Male Rescue (HMR) (29), which was initially identified as an allele mediating hybrid lethality of Drosophila melanogaster with sibling species (30) and is required to silence heterochromatic repeats (29,31). Although NLP has been found to localize to the centromere as well (25), molecular underpinnings of this localization are unknown.

Here, we set out to examine the functional role of NLP oligomerization for its localization at the centromere. We first characterize the oligomeric complexes formed by NLP and NPH and generate mutants which are unable to oligomerize. We find that these mutants fail to target to centromeres and to associate with HMR. Importantly, we demonstrate that HMR is required to recruit NLP oligomers to the centromere. Finally, we performed STED microscopy and could show that NLP and HMR domains largely co-localize with each other at centromere clusters but are distinct from the centromeric chromatin domains defined by dCENP-A.

## MATERIALS AND METHODS

### Cell culture

**Figure 1.**
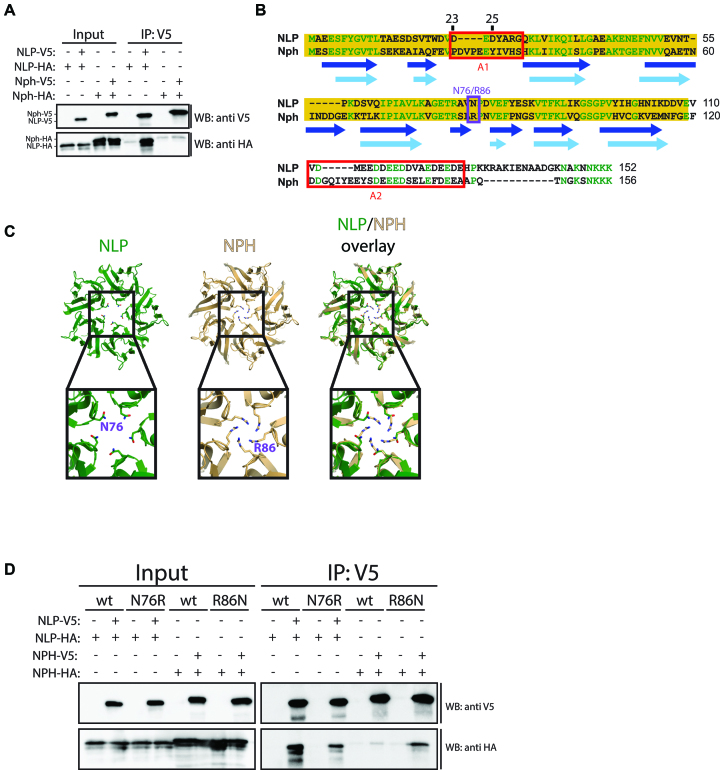
Self-oligomerization of NLP and NPH. (**A**) Schneider S2 cells transiently co-transfected with the indicated combinations of NLP-V5 and NLP-HA or NPH-V5 and NPH-HA were lysed and subjected to immunoprecipitation using V5 antibody. Immunoprecipitations were analysed by western blotting with αV5 and αHA antibodies. (**B**) Alignment of NLP and NPH amino acid sequence. Experimental secondary structures of NLP (taken from 13) and predicted secondary structures of NPH are indicated in dark and light blue, respectively. Secondary structure prediction was performed with PSIPRED v3.3. Identical amino acids are highlighted in green, the core domains are shown in yellow and the acidic stretches A1 and A2 with red boxes. Amino acid residues N76 on NLP and R86 on NPH are shown in the purple box. (**C**) Structural modelling of a NPH pentamer. (**D**) Schneider S2 cells transiently co-transfected with the indicated combinations of wt or N76R mutant NLP-V5/HA and wt or R86N NPH-V5/HA were lysed and subjected to immunoprecipitation using αV5 antibody. Immunoprecipitations were analysed by western blotting with αV5 and αHA antibodies.

**Figure 2.**
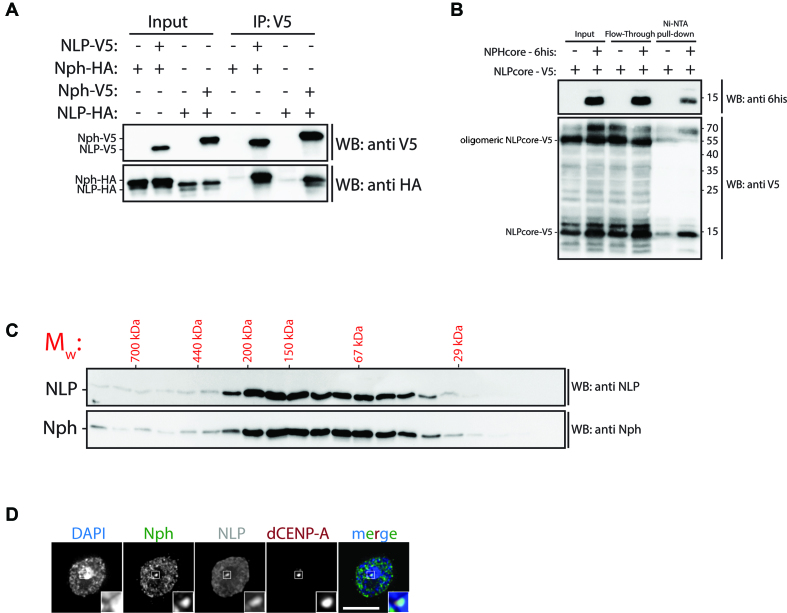
NLP and NPH hetero-oligomerize. (**A**) Schneider S2 cells transiently co-transfected with the indicated combinations of NLP-V5, NLP-HA, NPH-V5 and NPH-HA were lysed and subjected to immunoprecipitation using V5 antibody. Immunoprecipitations were analysed by western blotting with αV5 and αHA antibodies. (**B**) NPHcore-6his and NLPcore-V5 were co-expressed in bacteria, purified through Ni-NTA pull-down and analysed by western blotting with α6his and αV5 antibodies. (**C**) Total cell extracts prepared from Schneider S2 cells were run on size exclusion chromatography column and fractions analysed by western blotting with αNLP and αNPH antibodies. (**D**) Schneider S2 cells were stained with αNPH, αNLP and αdCENP-A antibodies and analysed by immunofluorescence microscopy. DAPI was used to visualize DNA. Insets show 3-fold magnification of boxed regions. Scale bar: 5μm.

Drosophila Schneider S2 cells were grown at 25°C in Schneider's Drosophila medium (Serva) supplemented with 10% Fetal Calf Serum (FCS) and antibiotics (0.3 mg/ml Penicillin, 0.3 mg/ml Streptomycin and 0.75 μg/ml Amphotericin B). For transfection of cells with plasmids, XtremeGene HP (Roche) was used. Cells were harvested 72 h post-transfection. In experiments shown in Figures 1A, D, 2A, 5A, B and 6A, B and Supplementary Figure S1B, the pMT promoter on the plasmids was induced with 500 μM CuSO_4_ 24 h post-transfection.

### Cloning

NLP F6E, I62E and V79E mutations were generated through overlap extension PCR and cloned into pMT vector. NLP F6E/I62E/V79E triple mutation, NLP N76R, NPH R86N, NLP A1 mutant and NLP tail mutant were synthesized by Integrated DNA Technologies (IDT) and cloned into required vectors. Details on all plasmid constructions are available upon request.

### Antibodies

Antibodies against Nph were produced at the Helmholtz-Zentrum München by injecting rats with the recombinant NPHcore-6his domain. Two hybridoma clones were isolated (1E8 and 9F11).

**Table:** 

Epitope	Species	Concentration	Origin
6xHis	Rabbit	WB: 1:1000	Cell Signaling, #2365
Nph	Rat	IF: undiluted	Clone #9F11, produced during this study
Nph	Rat	WB: 1:100	Clone #1E8, produced during this study
dCENP-A	Chicken	IF: 1:100	Clone H31, Heun
		STED: 1:25	Lab
HA	Rabbit	IF: 1:100	Santa Cruz, sc-805
HA	Mouse	WB: 1:10000	Clone 12CA5, kind gift from Dr. Simona Saccani (IRCAN, Nice)
HMR	Rat	WB: 1:25	Clone 2C10,
		IF: 1:15	kind gift from
		STED: 1:5	Prof. Axel Imhof (LMU Munich)
NLP	Rabbit	WB: 1:1000	Padeken *et al.*,
		IF: 1:100	2013
		STED: 1:25	
NLP	Rat	STED: 1:25	Padeken *et al.*, 2013
Tubulin	Mouse	WB: 1:1000	DSHB, AA4.3
V5	Rabbit	IP: 1μl per sample	Sigma, V8137
		WB: 1:1000	
V5	Mouse	WB: 1:1000	Invitrogen,
		IF: 1:100	R96025

### Immunoprecipitations

**Figure 3.**
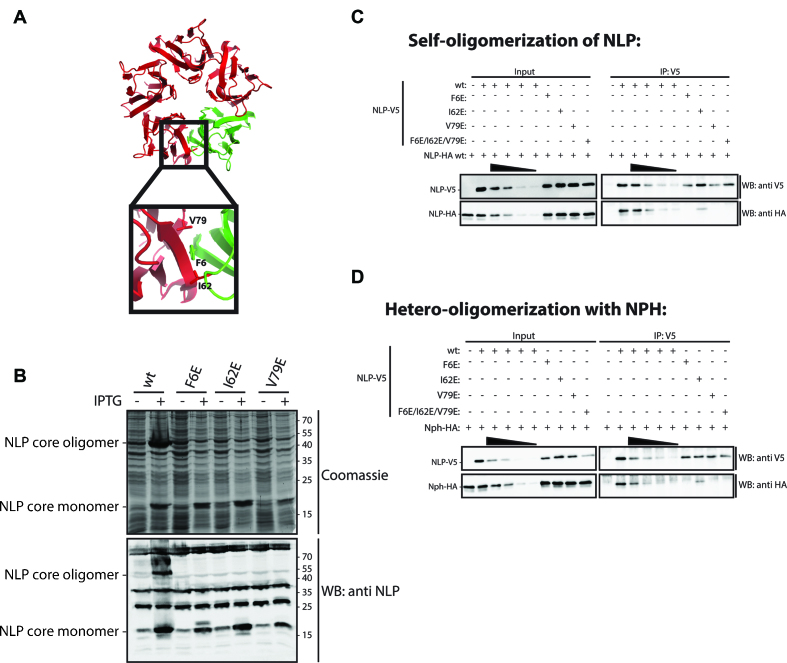
Generation of oligomerization-deficient NLP mutants. (**A**) Crystal structure of the NLP pentamer (13). Indicated are three amino acids (F6, I62, V79) at the hydrophobic interphase between NLP subunits. (**B**) Lysates of bacteria expressing the wt NLP core domain or NLP core domain carrying the mutations F6E, I62E and V79E were run on SDS-PAGE and analysed with Coomassie staining or western blotting with αNLP antibodies. (**C**) Schneider S2 cells transiently co-transfected with the indicated combinations of wt or mutant NLP-V5 and wt NLP-HA were lysed and subjected to immunoprecipitation using αV5 and αHA antibodies. Immunoprecipitations were analysed by western blotting with the indicated antibodies. (**D**) Schneider S2 cells transiently co-transfected with the indicated combinations of wt or mutant NLP-V5 and wt NPH-HA were lysed and subjected to immunoprecipitation using αV5 antibody. Immunoprecipitations were analysed by western blotting with αV5 and αHA antibodies.

Cells were harvested, washed 2× in PBS and resuspended in hypotonic buffer (20 mM HEPES pH 7.9, 20 mM NaCl, 5 mM MgCl_2_, 1 mM PMSF, 1 mM DTT, cOmplete^™^ EDTA-free Protease Inhibitor Cocktail (Roche)) and incubated on ice for 10 min. Subsequently, cells were dounced with a 26$\frac{1}{2}$ G needle and again incubated on ice for 10 min. Nuclei were pelleted at 500 g, 5 min, 4°C and lysed in hypotonic buffer supplemented with 0.5% IGEPAL^®^ CA-630 (Sigma). To the lysate, Benzonase (Millipore) was added and rotated at 4°C for 1 h. Subsequently, NaCl concentration was raised to 300 mM through addition of 5M NaCl to lysate and rotated at 4°C for another 30 min. NaCl concentration was lowered back to 150 mM through addition of hypotonic buffer supplemented with 0.5% IGEPAL^®^ CA-630 and lysate cleared by centrifugation (15 000g, 15 min, 4°C). An aliquot of the sample was kept as input. For experiments shown in Figures 1A, D, 2A and 3C, D, rabbitαV5 antibody was added to the samples. Next day, Protein A Dynabeads pre-equilibrated in hypotonic buffer supplemented with 0.5% IGEPAL^®^ CA-630 and NaCl concentration of 150 mM was added to the samples and incubated rotating for 3 h at 4°C. Beads were extensively washed with hypotonic buffer supplemented with 0.5% IGEPAL^®^ CA-630 and NaCl concentration of 150 mM and bound proteins eluted with Lämmli Buffer at 95°C. For experiments shown in Figures 5A, B and 6A, B 50 μl Anti-V5 Agarose Affinity Gel (Sigma) per sample were pre-equilibrated in hypotonic buffer supplemented with 0.5% IGEPAL^®^ CA-630 and NaCl concentration of 150 mM and incubated with the sample rotating o/n at 4°C. Next day, beads were extensively washed with hypotonic buffer supplemented with 0.5% IGEPAL^®^ CA-630 and NaCl concentration of 150 mM and bound proteins eluted with Lämmli Buffer at 95°C. Inputs and IPs were loaded on 8% or 10% gels for HMR and 15% gels for NLP or NPH and blotted on Amersham Protran 0.2 NC nitrocellulose membranes. Blots were blocked in 5% milk/PBS–Tween or 5% BSA/PBS–Tween and incubated with indicated antibodies.

### Pull-down of NLP core and NPH core domains co-expressed in bacteria

For co-expression of NPHcore-6his and NLPcore-V5 shown in Figure 2B, BL21(DE3) bacteria were transformed with a pACYCDuet™-1 plasmid containing both cDNAs for NLPcore-V5 and NPHcore-6his or NLPcore-V5 only and expression induced with 0.5 mM IPTG at 37°C for 3 h. Bacteria were resuspended in lysis buffer containing 20 mM Tris–HCl pH 8, 150 mM NaCl, 0.5 β-mercaptoethanol, 10 mM Imidazole and cOmplete^™^ EDTA-free Protease Inhibitor Cocktail (Roche) and lysed through sonication. Lysates were then incubated with HIS-Select^®^ HF Nickel Affinity Gel (Sigma-Aldrich) for 1 h rotating at 4°C. Beads were extensively washed with lysis buffer and bound proteins eluted with Lämmli buffer at 95°C.

### Immunofluorescence

Cells were settled on polylysine-coated glass slides for 20 min and fixed with 3.7% formaldehyde (Sigma) in 0.1% Triton X-100/PBS for 10 min. Fixation solution was washed off for 5 min with 0.1% Triton X-100/PBS and slides blocked with Image-iT FX signal enhancer (Invitrogen) for 1 h. Staining with primary antibody was performed o/n at 4°C. Next day, slides were washed 3× 5 min with 0.1% Triton X-100/PBS and incubated with secondary antibodies coupled to Alexa fluorophores for 1h at room temperature (RT). Slides were again washed 3× 5 min with 0.1% Triton X-100/PBS and incubated with DAPI for 3 min. Excess DAPI was washed off with 0.1% Triton X-100/PBS for 5 min and samples mounted with SlowFade Gold (Invitrogen).

**Figure 4.**
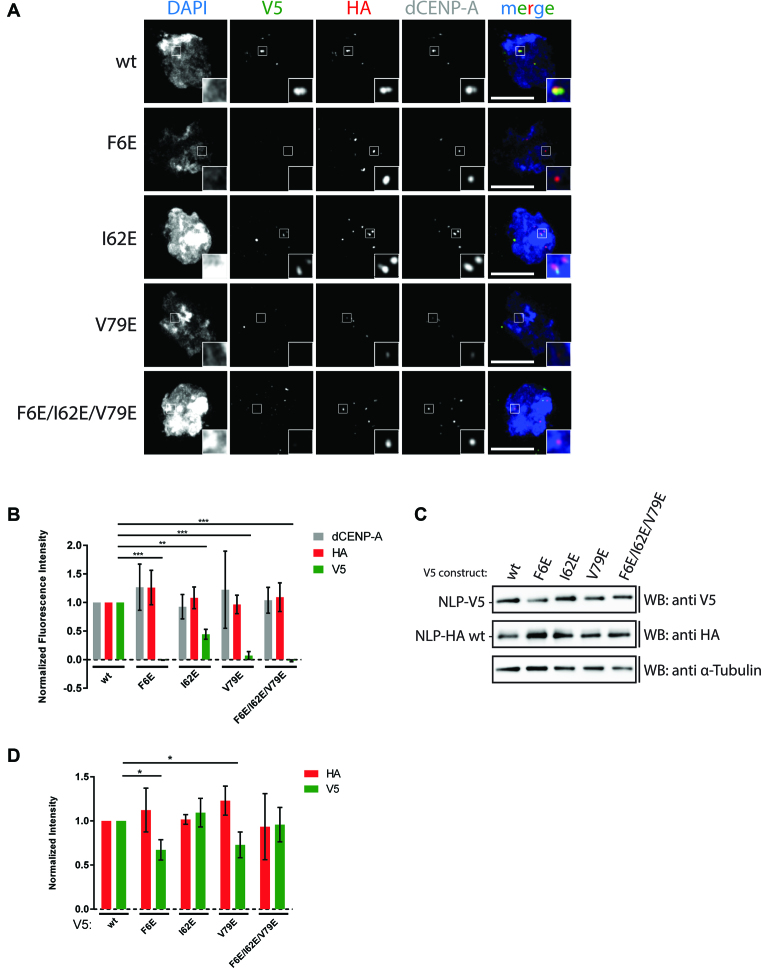
Oligomerization-deficient NLP mutants fail to localize to the centromere. (**A**) Schneider S2 cells were transiently co-transfected with wt or mutant NLP-V5 and wt NLP-HA constructs, cytospun and analysed by immunofluorescence microscopy with αV5, αHA and αdCENP-A antibodies. DAPI was used to visualize DNA. Insets show 3-fold magnification of boxed regions. Scale bars: 5 μm. (**B**) Quantification of (A). Graph shows mean of 3 independent experiments. In each experiment, at least 20 cells were quantified per condition. Error bars: SD. *P* values were calculated with unpaired *t*-test and are represented as follows: *P* ≤ 0.05 by *, ≤0.001 by ** and ≤0.0001 by ***. Comparisons between wt and mutants with *P* values >0.05 were considered not significant and are not indicated in the graphs. (**C**) Cells as in (A) were lysed and analysed by western blotting with αV5, αHA and αα-Tubulin antibodies. (**D**) Densitometric analysis of (C). Graph shows mean of 3 independent experiments. Error bars: SD. *P* values were calculated with unpaired t-test and are represented as follows: *P* ≤ 0.05 by *, ≤0.001 by ** and ≤0.0001 by ***. Comparisons between wt and mutants with *P* values >0.05 were considered not significant and are not indicated in the graphs.

**Figure 5.**
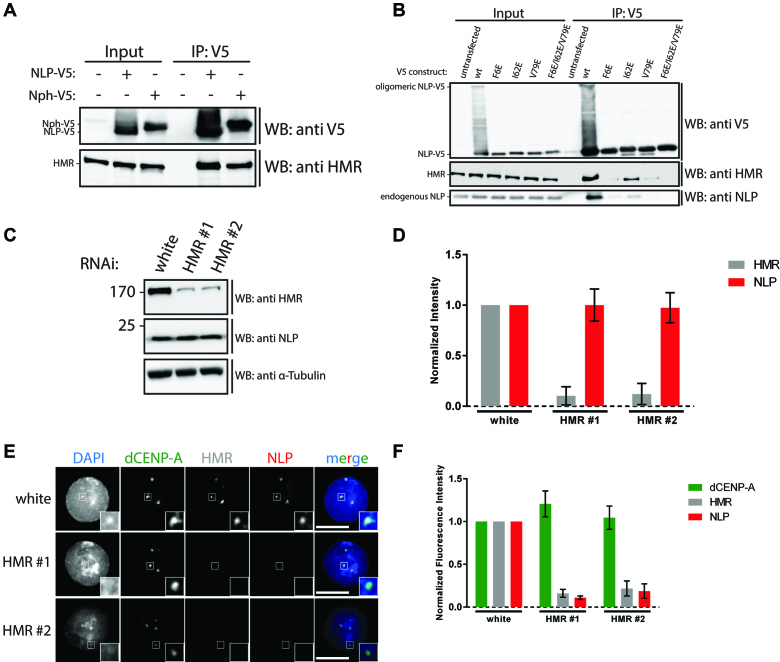
NLP is recruited to the centromere by HMR. (**A**) Schneider S2 cells transiently transfected with NLP-V5 or NPH-V5 were lysed and subjected to immunoprecipitation using αV5 antibody. Immunoprecipitations were analysed by western blotting with αV5 and αHMR antibodies. (**B**) Schneider S2 cells transiently transfected with wt or indicated mutant NLP-V5 were lysed and subjected to immunoprecipitation using αV5 antibody. Immunoprecipitations were analysed by western blotting with αV5, αHMR and αNLP antibodies. (**C**) Schneider S2 cells incubated with dsRNA against white or HMR were lysed and analysed by western blotting with αHMR, αNLP and αα-Tubulin antibodies. (**D**) Densitometric analysis of (C). Graph shows mean of 3 independent experiments. Error bars: SD. (**E**) Cells as in (C) were cytospun and analysed by immunofluorescence microscopy with αdCENP-A, αHMR and αNLP antibodies. DAPI was used to visualize DNA. Insets show 3-fold magnification of boxed regions. Scale bars: 5 μm. (**F**) Quantification of (E). Graph shows mean of three independent experiments. In each experiment, 20 cells were quantified per condition. Error bars: SD.

On settled cells, the high abundance of NLP and NPH in the nucleoplasm makes it difficult to visualize their centromeric signal. For better visualization of the centromeric signal, the nucleoplasmic pool can be removed through prelysis (Supplementary Figure S2B) or cytospin (Figures 4A and 5E). For prelysis, cells were settled on slides, incubated with 0.1% Triton X/PBS for 30sec-1min, washed with PBS for 1 min and then fixed in 3.7% formaldehyde in PBS for 10 min and processed as described above for settled cells. For cytospin, cells were harvested, resuspended in 500 μl 0.5% sodium citrate and incubated for 10 min at RT. Subsequently, samples were spun on a polylysine-coated glass slide in a Shandon Cytospin 4 for 10 min at 900 rpm (high acceleration). Slides were then fixed in 3.7% formaldehyde in PBS for 10 min and processed as described above for settled cells. For experiment shown in Figure 4A, 2 × 10^5^ cells were used for each condition. The release of nucleoplasmic NLP makes it difficult to judge whether a cells has been transfected. Thus, in the experiment shown in Figure 4A, we co-transfected a wt NLP-HA construct, which served as an internal control to select only transfected cells. To analyze the samples in an unbiased manner, we screened for cells where wt NLP-HA was detectable at the centromere and then imaged these cells for quantification, being blind for the presence or absence of wt or mutant NLP-V5 at the centromere. For the experiment shown in Figure 5E, 5 × 10^4^ cells were used per condition. Cells which showed bright dCENP-A staining and no or very low nucleoplasmic signal for NLP were selected for quantification. These cells were usually found in areas of the slide with a low density of cells.

### RNAi

Double stranded RNAs targeting white or HMR were produced through *in vitro* reverse transcription of PCR products using T7 polymerase. The PCR products were generated with forward and reverse primers containing the T7 promoter sequences at their 5′ end. 1 × 10^6^ cells were plated in 1 ml medium in a well of a six-well plate and the next day, medium was replaced with 1ml serum free medium containing 20 μg of dsRNA. After incubation for 30 min, 3 ml medium containing serum was added. Cells were harvested 6 days after treatment with the dsRNA and processed for Western Blot or Immunofluorescence.

The following primers were used for PCR:

**Table:** 

Primer	Sequence
white FW	TTAATACGACTCACTATAGGGACTGCTCAATGGCCAACCTGTGGA
white RV	TTAATACGACTCACTATAGGGCCTCGGCCATCAGAAGGATCTTGT
HMR#1 FW	TTAATACGACTCACTATAGGGAGAGATTGCCAGATAATTGTTTCTGACACAATTGGTC
HMR#1 RV	TTAATACGACTCACTATAGGGAGATCGCTCTCATCCACATGCTCTTTTTCAAG
HMR#2 FW	TTAATACGACTCACTATAGGGAGAGATGTGGAGGTCATAGAGAATCCGCCAATG
HMR#2 RV	TTAATACGACTCACTATAGGGAGAACCTTGTTGTGCAGGGAGTCCTCCGTC

### Size exclusion chromatography of total cell extracts

Cells were washed 3× in PBS, resuspended in lysis buffer (50 mM Tris–HCl pH 8, 150 mM NaCl, 1 mM EDTA, 1% reduced Triton X-100, 1 mM MgCl_2_, 0.5 mM PMSF, cOmplete^™^ EDTA-free Protease Inhibitor Cocktail), sonicated and lysate cleared by centrifugation. Lysate where then filtered first through Millipore Ultrafree^®^ MC-HV 0.45 μm and then through Millipore Ultrafree^®^ MC-GV 0.22 μm. Sample was run in the Edinburgh Protein Production Facility on a Superdex200™ 10/300 GL column (GE Healthcare) pre-equilibrated in lysis buffer w/o protease inhibitor cocktail.

### Protein expression and purification

For injection into rats and antibody production, the 6his-tagged NPHcore was expressed in bacteria and bacteria pelleted, washed 2× with PBS and resuspended in lysis buffer (20 mM Tris–HCl pH8, 150 mM NaCl, 0.5 mM β-mercaptoethanol, 10 mM imidazole, cOmplete^™^, EDTA-free Protease Inhibitor Cocktail (Roche)) and lysed by sonication. Lysates were cleared through centrifugation and bound to a HiTrap™ Chelating HP column (GE Healthcare) pre-equilibrated with lysis buffer. Column was washed with wash buffer (20 mM Tris–HCl pH 8, 150 mM NaCl, 0.5 mM β-mercaptoethanol, 30 mM imidazole) and eluted with elution buffer (20 mM Tris–HCl pH 8, 150 mM NaCl, 0.5 mM β-mercaptoethanol, 300 mM imidazole). Fractions containing the protein of interest were pooled and dialyzed against buffer containing 50 mM Tris–HCl pH 8 and 150 mM NaCl.

For experiment shown in Supplementary Figure S1A, NLPcore-6his and NPHcore-6his were expressed in BL21(DE3) and expression was induced with IPTG at 37°C for 3 h. Purification was performed essentially according to a protocol described in The QIAexpressionist™ handbook (Qiagen). Pellets were washed with PBS and resuspended in denaturing buffer (100 mM NaH_2_PO_4_, 10 mM Tris Base and 8 M urea) at pH 8 and stirred at RT for 1 h. Lysate was cleared by centrifugation and bound to a HiTrap™ Chelating HP column (GE Healthcare). Column was washed with denaturing buffer at pH 6.3. Proteins were first eluted with denaturing buffer at pH 5.9, then denaturing buffer at pH 4.5. Fractions containing protein were pooled and dialysed against dialysis buffer (20 mM Tris–HCl pH 8, 200 mM NaCl, 10% glycerol, 0.5 mM β-mercaptoethanol) for refolding and refolded proteins were isolated through size exclusion chromatography on a Superdex200 10/300 GL (GE Healthcare).

### Microscopy

Images were acquired on a DeltaVision RT Elite Microscope and deconvolved and quick-projected using SoftWorX Explorer Suite^®^ (Applied Precision). Images were cropped in Adobe Photoshop and arranged in Adobe Illustrator.

### Quantifications and statistical analysis

Quantification of centromeric fluorescence intensities was performed on quick-projected images using ImageJ 1.50b. In every cell, a circle of constant size was placed around each centromere and mean intensity was measured. To account for background fluorescence, the intensity at three random non-centromeric locations within the same cell was measured and the average intensity of these three areas calculated. This value was then substracted from the measured centromeric fluorescence intensities (corrected centromere intensities). All corrected centromere intensities within one condition were summed up and the average calculated.

For quantification of western blots, Image Lab 5.2 (Bio-Rad) was used. A rectangle of constant size was placed around the bands and the value displayed under ‘Adj. Vol (Int)’ taken. For quantifications shown in Figures 4D and 5D, measured values were first normalized to α-Tubulin and then to the control sample. All Graphs were prepared in GraphPad Prism 7.02.

### STED microscopy

For STED microscopy, cells were grown on polylysine coated coverslips and samples were prepared with prelysis as described above, and mounted using ProLong™ Diamond (Life Technologies).

STED and confocal images were recorded at the Core Facility Bioimaging at the Biomedical Center, LMU Munich. Gated STED images were acquired with a Leica TCS SP8 STED 3X microscope with pulsed white light laser excitation and pulsed depletion with a 775 nm laser using an HC PL APO CS2 100×/1.40 oil immersion objective. The fluorescence was recorded line sequentially at a scan speed of 200 Hz, a pinhole setting of 0.93 AU (at 580 nm) and the pixel size was set to 25 nm × 25 nm; *z*-step size of z-stacks was 160 nm. The signals were detected with hybrid detectors operated in photon counting mode with the time gate set to 0.5–8 ns and using the following settings:

Alexa Fluor 594: excitation 590 nm; emission: 600–625 nm; depletion power: 50%.

Abberior STAR 635P: excitation 635 nm; emission: 643–720 nm; depletion power: 25%.

Images were deconvolved with Huygens Professional (SVI) and processed in Leica Application Suite X 3.3.0.16799.

## RESULTS

### Self- and hetero-oligomerization of NLP and NPH

Oligomerization of NPM1 in human cells has been found to be important for several aspects of its function, including nucleolar localization and nucleosome assembly (17–20). Similar to NPM1 and other nucleoplasmin homologues, the NLP crystal structure revealed a pentameric assembly and oligomers were detected after chemical crosslinking of embryonic extracts (13,22). To investigate NLP self-oligomerization *in vivo*, we transfected Schneider S2 cells with differently tagged versions of NLP, NLP-V5 and NLP-HA, and assayed their interaction by immunoprecipitation (IP). We found that NLP-V5 co-IPs with NLP-HA, thus showing that NLP is able to self-oligomerize *in vivo* (Figure 1A). The purified NLP core domain forms an oligomer which is thermostable and detectable on SDS-PAGE around 45 kDa (Supplementary Figure S1A) (13). Similarly, when overexpressed in S2 cells, NLP forms a high-molecular weight species which is detectable on western blots, likely reflecting the presence of pentamers *in vivo* (Supplementary Figure S1B).

Surprisingly, unlike for NLP, for its paralog NPH we could detect no or only very weak binding to itself using two differently tagged constructs (Figure 1A and D). In addition, no thermostable high-molecular weight species of NPH-V5 was visible on western blot after overexpression in S2 cells (Supplementary Figure S1B). To explore a possible explanation for this observation, we used PSIPRED to predict secondary structures on NPH. We find that the N-terminus of NPH contains predicted β-sheets, which largely overlap with the experimentally determined secondary structures of NLP (Figure 1B). In contrast, the C-terminus of NPH is predicted to be devoid of any secondary structure. Thus, NPH has an organization into ‘core’ and ‘tail’ domain similar to NLP (Figure 1B). In the absence of an experimental NPH structure, we generated a homology model of a hypothetical oligomeric assembly of NPH based on the crystal structure of NLP using the web-based homology modelling server Phyre2 (http://www.sbg.bio.ic.ac.uk/∼phyre/, Figure 1C). While the NPH pentamer reveals apolar interactions between the protomers as NLP *in silico*, the model predicts that a positively charged arginine would be exposed into the central cavity of the NPH pentamer. This would likely result in electrostatic repulsion, making a higher NPH oligomer unstable. To test this hypothesis, we mutated asparagine 76 in NLP to arginine (NLP N76R) and arginine 86 in NPH to asparagine (NPH R86N) and tested their ability to self-oligomerize (Figure 1D). In line with our hypothesis, mutant NPH R86N now shows robust self-oligomerization, while NLP N76R shows a minor effect on self-oligomerization (Figure 1D).

We next examined whether NLP and NPH can hetero-oligomerize. An interaction between the two proteins has been detected by previous proteome-wide interaction studies in *Drosophila* (32,33). When V5-tagged NLP was co-transfected with HA-tagged NPH or *vice versa* into S2 cells, we could readily observe an interaction between NLP and NPH by co-IP (Figure 2A). In addition, when co-expressed in bacteria the recombinant core domains of both proteins are sufficient to directly interact with each other (Figure 2B). To gain further insight into the oligomerization of NLP and NPH, we performed size exclusion chromatography (SEC) of total cell extracts (Figure 2C) and find that both proteins elute in fractions that correspond to a broad range of molecular weights. Importantly, all of these fractions contain always both proteins, NLP and NPH, further supporting the formation of hetero-oligomeric complexes (Figure 2C). To visualize the localization of NPH, we transfected S2 cells with NPH-V5 and found its localization highly reminiscent to NLP, namely nuclear but excluded from the nucleolus (Supplementary Figure S2A). The high abundance of NLP and NPH in the nucleoplasm often renders it difficult to visualize their localization to specific chromatin domains. Prelysis of the cells before fixation releases this nucleoplasmic pool and revealed that NPH-V5 localizes to the centromere, as observed for NLP-V5 (25) (Supplementary Figure S2B). Similarly, using an antibody specific for NPH, we were able to co-localize endogenous NPH with NLP in the nucleoplasm and at the centromere (Figure 2D and Supplementary Figure S1A, B). Like NLP (25), we find NPH to be absent from the centromere in mitosis (Supplementary Figure S2C). Together, this data suggests that NLP and NPH reside within the same complexes *in vivo*, most likely through the formation of a hetero-pentamer.

### Oligomerization of NLP is required for its localization at the centromere

To identify residues critical for oligomerization in NLP, we used the published crystal structure of NLP (13). The interface between two subunits within the NLP pentamer is comprised of hydrophobic residues (13). Three of these residues, F6, I62 and V79 were therefore mutated to charged glutamates (F6E, I62E and V79E, respectively, Figure 3A) and the resulting NLP core mutants tested for oligomer formation when expressed in bacteria (Figure 3B). In agreement with previous reports (13), we found the wt NLP core oligomer to be highly thermostable and can be detected on SDS-PAGE (Figure 3B). In contrast, none of the NLP core mutants F6E, I62E and V79E displayed oligomer formation in this assay, although all of them were expressed at comparable levels (Figure 3B). To test their ability to form oligomers *in vivo*, we transfected V5-tagged mutant NLP and probed their interaction with HA-tagged wt NLP. While the I62E mutant shows low but detectable interaction with wt NLP-HA, the F6E, V79E and a F6E/I62E/V79E triple mutant abolish self-oligomerization of NLP (Figure 3C). We also analyzed the ability of these mutants to hetero-oligomerize with NPH and find a similar interaction pattern (Figure 3D). Thus, while the I62E retains the ability to oligomerize *in vivo* to some extent, all other NLP mutants lose their ability to oligomerize with either NLP or NPH.

To test whether oligomerization-deficient NLP localizes to the centromere, we expressed the mutants with a V5-tag in S2 cells. As an alternative to prelysis and more reproducible, we cytospun the transfected cells on a microscopy slide, which similarly leads to a release of nucleoplasmic NLP from the nucleus. All V5-tagged oligomerization-deficient NLP mutants were co-transfected with a HA-tagged wt NLP construct, which serves as an internal positive control to identify transfected cells. Strikingly, all mutants that are unable to form oligomers were completely absent from the centromere (Figure 4A, B), despite being imported into the nucleus and having similar expression levels (Supplementary Figure S3 and Figure 4C, D). Consistent with its ability to oligomerize to a small extent, the I62E mutant shows low but detectable levels of localization to the centromere (Figure 4A, B). Thus, the ability of NLP to oligomerize is a pre-requisite for its localization to the centromere.

### NLP oligomers are recruited to the centromere by HMR

The impaired localization of oligomerization-deficient NLP to the centromere most likely reflects a down-stream consequence of its inability to associate with a centromeric interaction partner.

Previously, mass-spectrometric analysis of IPs of the protein ‘Hybrid Male Rescue’ (HMR) has revealed both NLP and NPH as potential interaction partners (29). Importantly, the same study also found HMR to localize to the centromere (29). In agreement with the previous report, we could verify the interaction between NLP, NPH and HMR through the co-IP of V5-tagged NLP or NPH with endogenous HMR (Figure 5A). We next tested the binding of oligomerization-deficient NLP to HMR, Interestingly, the oligomerization-deficient NLP mutants F6E, V79E and F6E/I62E/V79E failed to interact with HMR as judged by co-IP, while the mutant I62E showed low but detectable levels of interaction with HMR (Figure 5B). Thus, oligomerization of NLP is required to interact with HMR. This finding also establishes a correlation between the ability of NLP to oligomerize, localize to the centromere and to interact with HMR. To explore whether these three findings are causative, we investigated whether HMR is required for the localization of NLP to the centromere. S2 cells were treated with two different dsRNA oligonucleotides, which depleted HMR (Figure 5C, D), while not affecting protein levels of NLP (Figure 5C, D) or its import into the nucleus (Supplementary Figure S4). Importantly, we find that NLP failed to localize to the centromere in HMR depleted cells, indicating that HMR is required for the localization of the NLP oligomer to the centromere (Figure 5E, F).

### Acidic residues in the tail domain of NLP are required to interact with HMR

**Figure 6.**
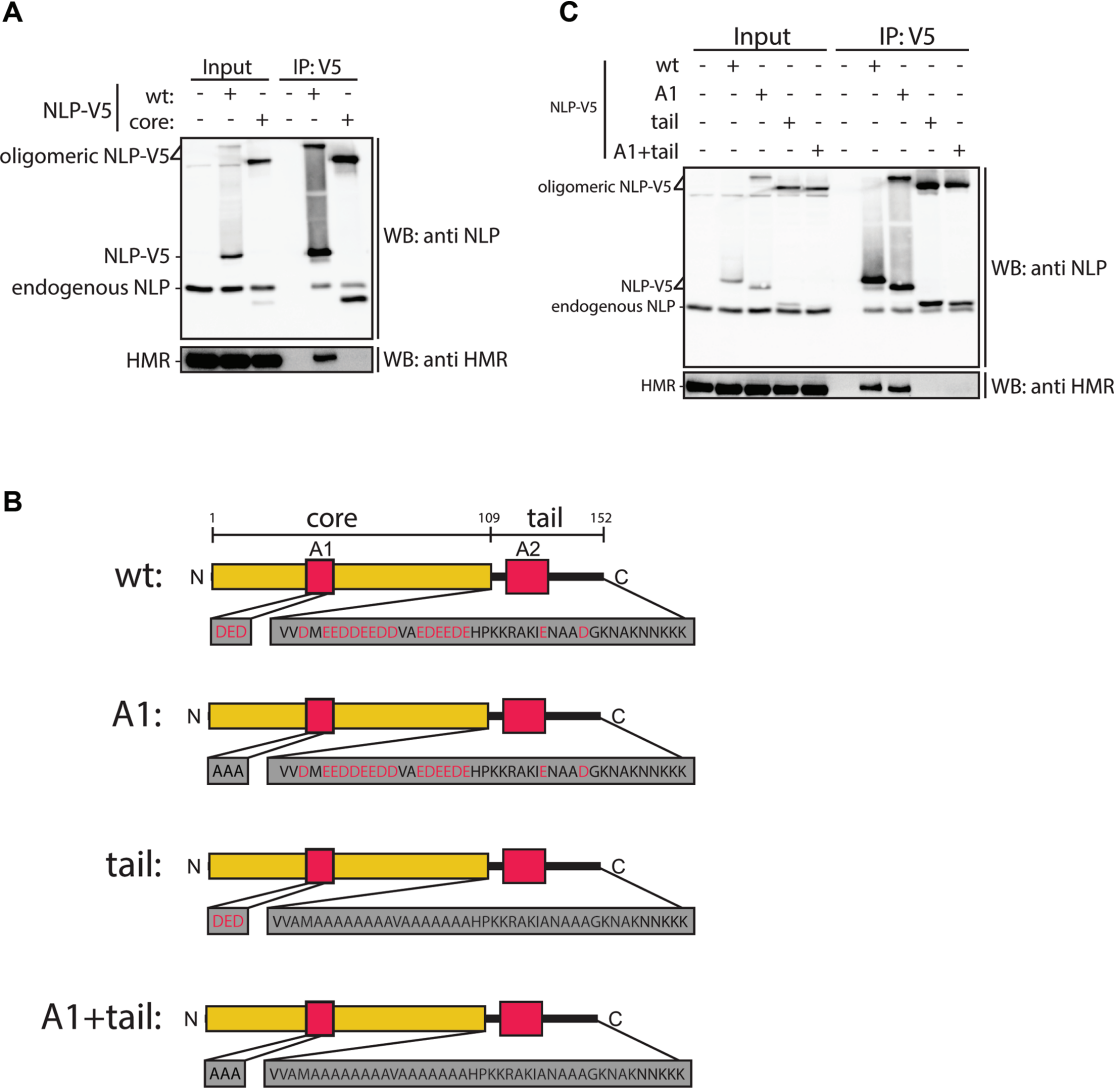
The acidic residues in the NLP tail domain are required for the interaction with HMR. (**A**) Schneider S2 cells transiently transfected with wt NLP-V5 or NLP core-V5 were lysed and subjected to immunoprecipitation using αV5 antibody. Immunoprecipitations were analysed by western blotting with αNLP and αHMR antibodies. (**B**) Constructs used for experiment shown in (C). The core domain is shown in yellow and the acidic stretches A1 and A2 with red boxes. Acidic residues shown in red were mutated to alanines shown in black. (**C**) Schneider S2 cells transiently transfected with wt or mutant NLP-V5 as indicated were lysed and subjected to immunoprecipitation using αV5 antibody. Immunoprecipitations were analysed by western blotting with αNLP and αHMR antibodies.

We next aimed to assess which regions of NLP mediate its interaction with HMR. Interestingly, despite the fact that the ability to oligomerize resides in the core domain, we found this domain of NLP to be insufficient for binding to HMR, implicating the tail domain as an additional element critical for this interaction (Figure 6A). A characteristic feature of the NLP tail domain is the presence of acidic residues which are mainly clustered in the acidic A2 tract (Figures 1B and 6B). To explore whether the interaction with HMR is mediated through the acidic residues in the tail domain, we generated NLP mutants in which all acidic residues in the tail domain were mutated to alanines (Figure 6B). For comparison, we also mutated the acidic residues in the A1 tract located in the NLP core domain either alone or in addition to the A2 tract mutations (Figure 6B). While mutations in the A1 tract did not affect binding to HMR, mutating the acidic residues in the tail domain completely impairs this interaction (Figure 6C). Thus, the acidic nature of the NLP tail is essential for the interaction with HMR.

### NLP and HMR localize adjacent to the dCENP-A domain

**Figure 7.**
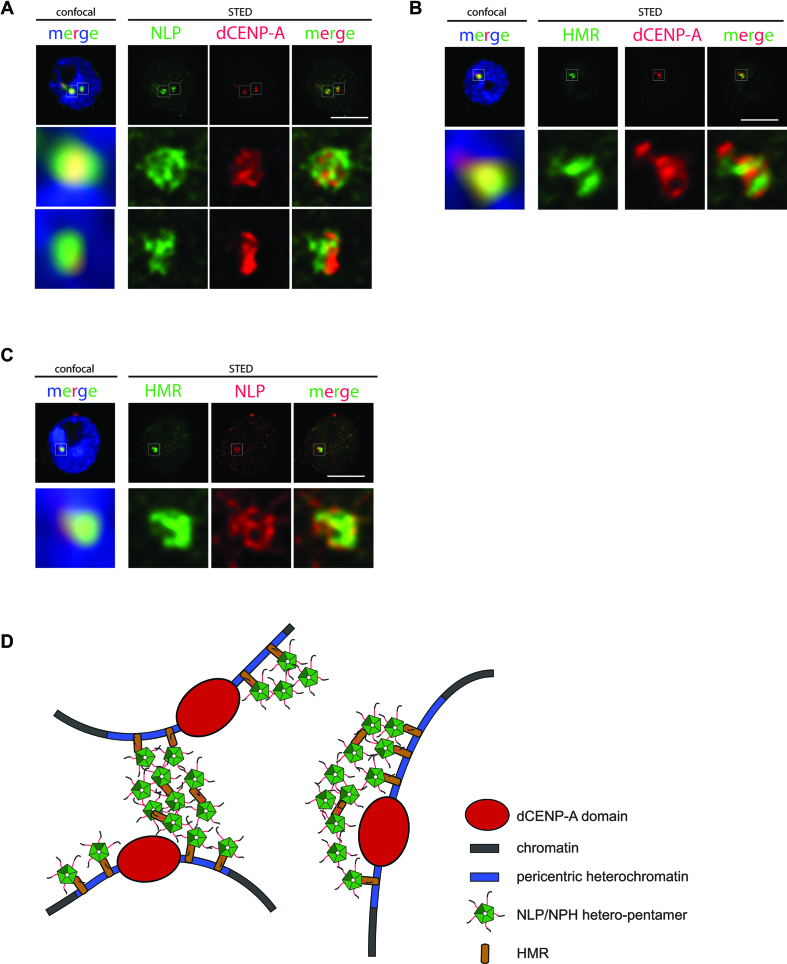
STED microscopy of NLP, HMR and dCENP-A. (**A**) Schneider S2 cells were prelysed and stained with (A) αNLP and αdCENP-A, (**B**) αHMR and αdCENP-A or (**C**) αHMR and αdNLP antibodies as indicated and were analysed by confocal and STED microscopy. DAPI was used to visualize DNA. Blow-ups show 7.3-fold magnification of boxed regions. Scale bars: 3 μm. (**D**) Model of the localization of NLP at the centromeric region. The NLP oligomer, possibly a NLP/NPH hetero-pentamer with 4:1 stoichiometry, is recruited to the centromere through HMR. Previously, HMR has been found to be enriched at pericentromeric regions and it has been hypothesized that HMR might not localize to the centromere core domain defined by dCENP-A, but rather to the flanking pericentric heterochromatin (38). NLP might form a network which fills the spaces in between the centromeric domains. Networks of NLP oligomers could be locally confined, span regions on the same chromosome or even between different chromosomes. Components in the scheme are not drawn to scale.

Having characterized the molecular mechanism of HMR dependent NLP targeting to the centromere, we aimed to obtain more detailed insights into the organization of both components in relation to the centromeric domain defined by dCENP-A. To this end we performed super-resolution microscopy using stimulated emission depletion (STED) microscopy, which enables imaging at a resolution of 30–80 nm (34) and allows the visualization of fine details within centromere clusters that are indiscernible using confocal microscopy (Figure 7). Strikingly, while NLP and dCENP-A seem to co-localize at low resolution, they appear as proximal but distinguishable domains in the STED images that share only limited spatial overlap (Figure 7A and Supplementary Figure S5A). In particular, locations with high density of dCENP-A often contain low levels of NLP and *vice versa*. We then co-stained HMR with dCENP-A and found that both, very similar to NLP and dCENP-A, show only partial overlap at high resolution (Figure 7B and Supplementary Figure S5B). In contrast, images of NLP and HMR show that although not identical in their localization pattern, display strong signal overlap even at high resolution (Figure 7C and Supplementary Figure S5C). Thus, NLP and HMR form domains, which are spatially separated from the centromeric domains defined by dCENP-A.

## DISCUSSION

The nucleoplasmin protein family shares a highly conserved N-terminal core domain responsible for oligomerization. In *Drosophila*, NLP forms pentamers *in vitro* and oligomers have been detected in embryonic extracts (13,22). Furthermore, proteome-wide interaction studies suggested an interaction between NLP and the second nucleoplasmin homologue in *Drosophila*, NPH (32,33). Here, we confirmed the interaction between NLP and NPH and show that the core domains of both proteins are sufficient to directly associate with each other. In addition, size exclusion chromatography indicates that complexes formed by NLP and NPH *in vivo* contain both proteins. Furthermore, we find that NLP can strongly co-IP with itself, unlike NPH, which suggests that homo-oligomers of the latter are unstable. Indeed, modelling of the NPH amino acid sequence onto the NLP pentamer structure revealed a cluster of charged arginine residues at the inner ring of a hypothetical NPH pentamer that would potentially lead to steric clashes and electrostatic repulsion. In agreement with this, an NPH R86N mutant shows robust self-oligomerization. Our data suggests the existence of hetero-oligomers formed by NLP and NPH *in vivo*, which contain multiple molecules of NLP, but likely only one NPH molecule. This is consistent with a 4:1 NLP-NPH stoichiometry *in vivo*, similar to what has been suggested for a complex consisting of human NPM1 and NPM3 *in vitro* (35). Although we do not yet know the physiological relevance why hetero-pentamers are formed, it is tempting to speculate that incorporating a different nucleoplasmin paralogue might help destabilize the otherwise highly stable homo-pentamer, thereby making it more amenable to regulatory mechanisms and disassembly. For instance, this could be required during mitosis, when NLP and NPH are removed from the centromeres (25).

To date the centromere is the only locus known to which NLP localizes, but the molecular underpinnings of this association are unknown. To understand the functional relevance of NLP oligomerization, we generated oligomerization-deficient NLP mutants and find that these mutants fail to localize to the centromere. Previously, mass-spectrometry of HMR immunoprecipitations has detected an interaction with NLP and NPH and we are able to confirm HMR co-IPs with both proteins. Interestingly, we find that oligomerization-deficient NLP mutants are unable to bind to HMR, suggesting a link with their inability to localize to centromeres. Indeed, depletion of HMR leads to loss of NLP from centromeres, demonstrating that the NLP oligomer is recruited to the centromere through interaction with HMR (Figure 5). Loss of NLP from centromeres has been found to cause centromere declustering (25). However, we did not observe centromere declustering after HMR RNAi ((29) and data not shown), a possibility being that the depletion of HMR is not complete and low levels of HMR and NLP remain at the centromeres. Apart from NLP, HMR has previously been found to interact with Lethal Hybrid Rescue (LHR) and tandem purifications of HMR and LHR contain both NLP and NPH (29). Interestingly, similar to NLP, LHR requires HMR for targeting to the centromere (29). How the interactions within this protein complex are mediated is currently not understood. We find that in addition to oligomerization of NLP, the acidic residues in the NLP tail domain are required for the interaction with HMR. Although it remains to be determined whether the interaction between NLP and HMR is direct, it is noteworthy that HMR has a theoretical isoelectrical point of 9.6, rendering it a basic, positively charged protein possibly capable of directly interacting with the acidic regions in NLP.

In human cells localization of NPM1 to the nucleolus has also been shown to require oligomerization (17,19,20). Mechanistically, NPM1 pentamers can interact with each other, which leads to liquid droplet formation of NPM1 and integration into the nucleolus (36,37). Interactions between NPM1 pentamers can be mediated through proteins containing arginine (R)-rich motifs or ribosomal RNA or through homotypic interactions between NPM1 tail domains (36,37). Phase separation of NPM1 through R-rich motif containing proteins requires the core domain of NPM1 as well as the acidic tract A2 in the tail domain (36). As NLP oligomerization is critical for its centromeric localization and oligomerization as well as the acidic residues in the A2 tract are required for its interaction with HMR, it is possible that the centromere association of NLP is mediated through a similar mechanism as NPM1 localization into the nucleolus. Consistent with the interaction between HMR and NLP, high resolution microscopy reveals that both largely co-localize at centromeres. Interestingly, neither protein shows much overlap with dCENP-A but rather fills the space in between centromeric foci, revealing the existence of distinct subdomains within the centromere cluster. It is tempting to speculate that these spaces are filled with interacting NLP oligomers. How this interaction might be mediated remains to be investigated but it could involve NLP molecules themselves, HMR or a yet unknown factor (Figure 7).

## Supplementary Material

Supplementary Data
