# Supplementary material for: Oligomerization of Drosophila Nucleoplasmin-Like Protein is required for its centromere localization
Source: Nucleic Acids Res. 2018 Oct 24;46(21):11274–86. doi: 10.1093/nar/gky988 (PMC6277087; doi:10.1093/nar/gky988)
Supplement: Supplementary Data [file gky988_supplemental_files.pdf]

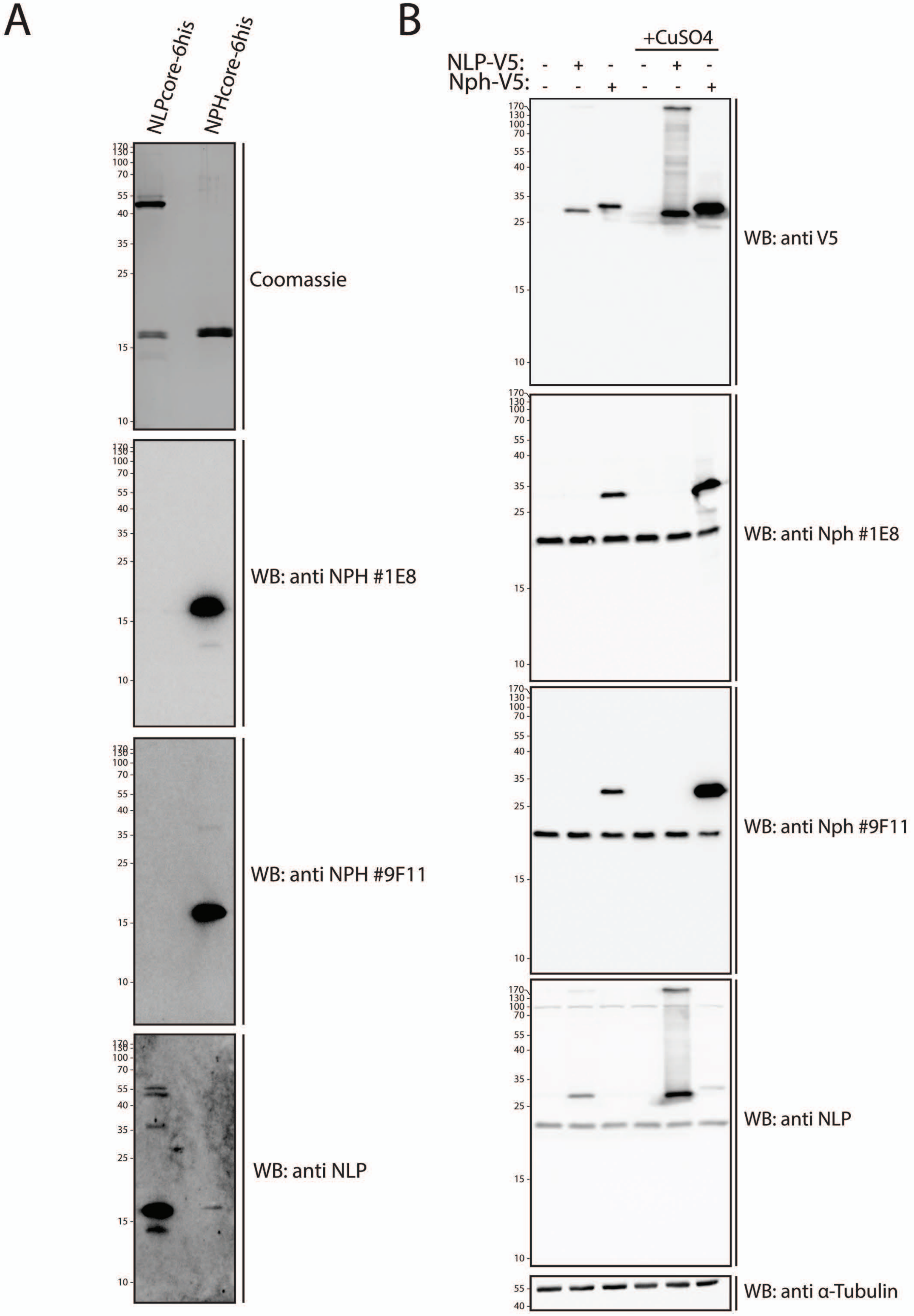

Figure S1: Characterization of monoclonal NPH antibodies

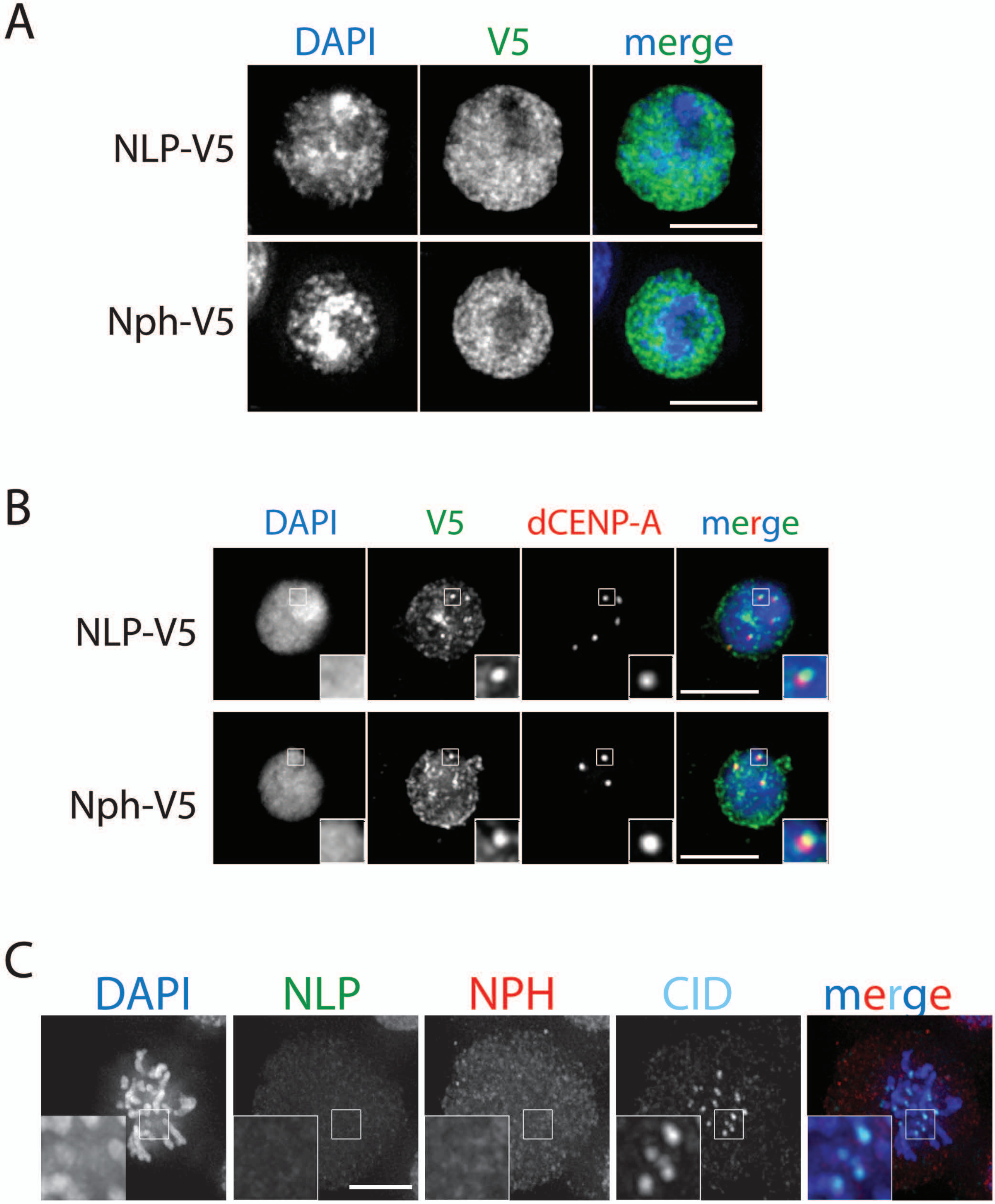

Figure S2: Localization of NLP and NPH

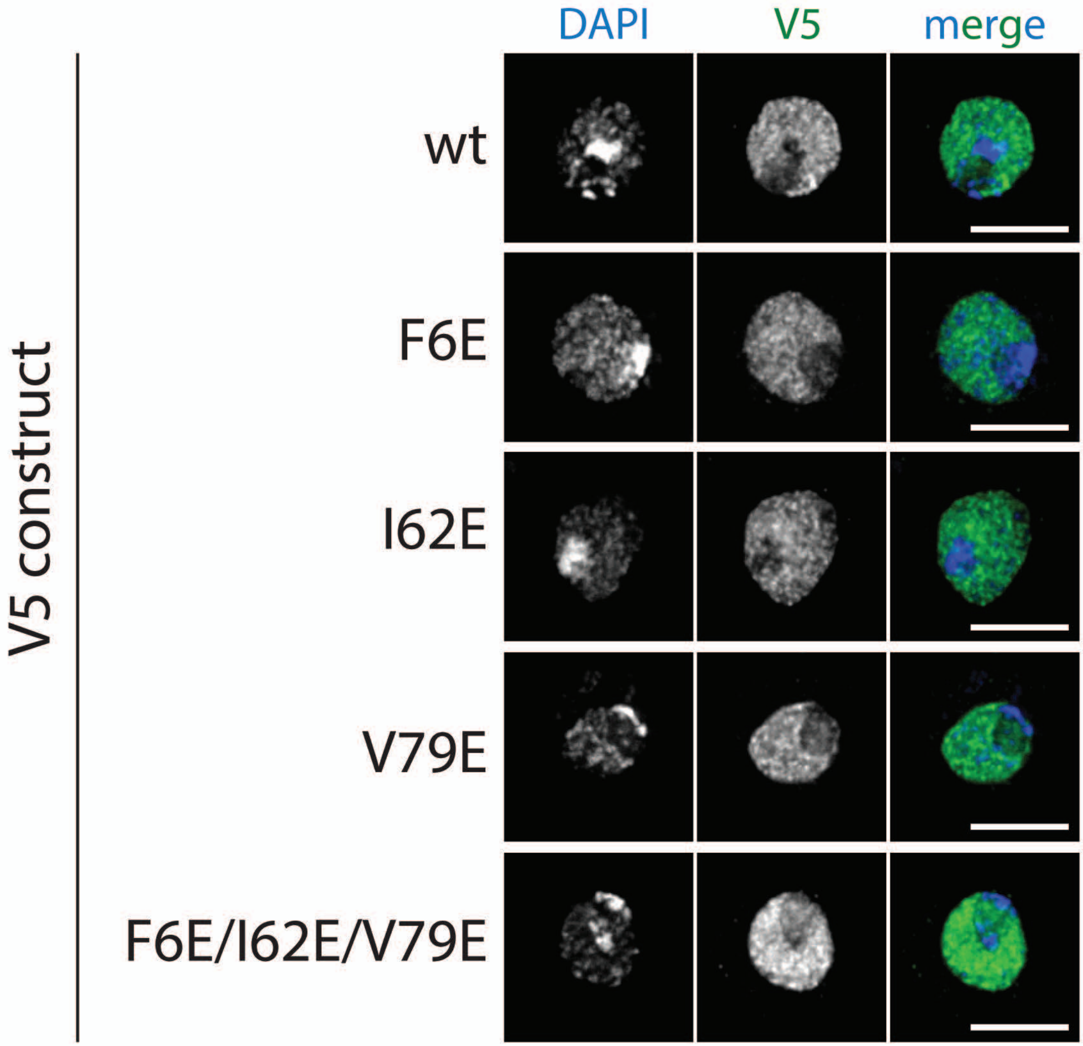

Figure S3: Oligomerization-deficient NLP mutants localize to the nucleoplasm

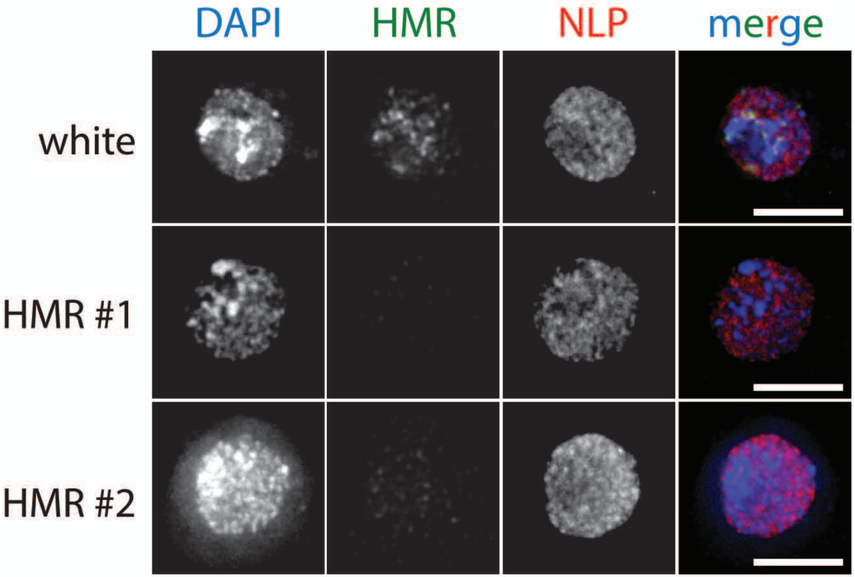

Figure S4: Nucleoplasmic NLP is not affected by the HMR RNAi

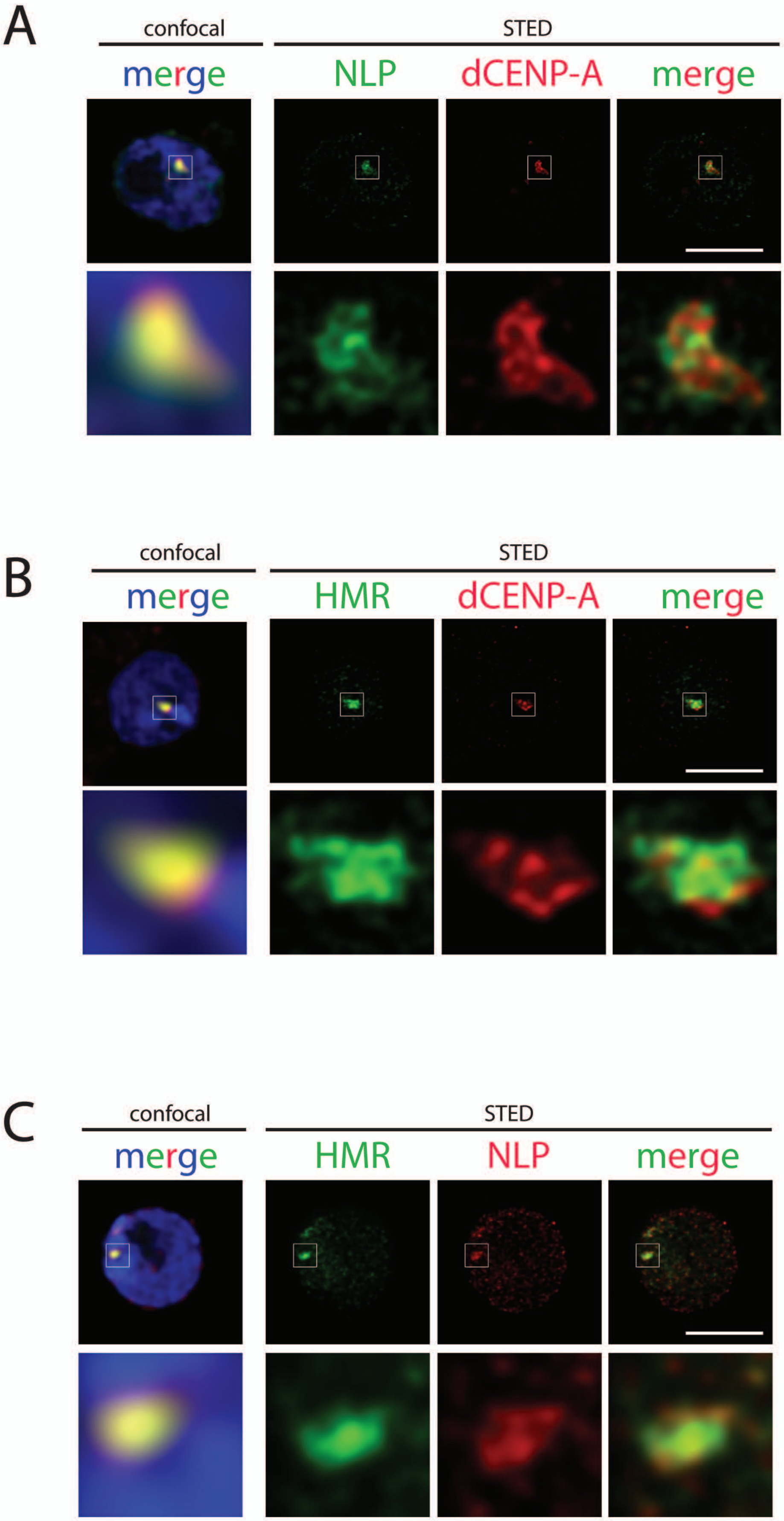

Figure S5: Additional STED images
